# Phytochemical profiling and anti-fibrotic activities of *Plumbago indica* L. and *Plumbago auriculata* Lam. in thioacetamide-induced liver fibrosis in rats

**DOI:** 10.1038/s41598-022-13718-9

**Published:** 2022-06-14

**Authors:** Nabil Mohamed Selim, Mina Michael Melk, Farouk Rasmy Melek, Dalia Osama Saleh, Mansour Sobeh, Seham S. El-Hawary

**Affiliations:** 1 Pharmacognosy Department, Faculty of Pharmacy, Cairo University, Giza, 12613 Egypt; 2 Chemistry of Natural Compounds Department, National Research Centre, Giza, 12622 Egypt; 3 Pharmacology Department, National Research Centre, Giza, 12622 Egypt; 4 AgroBioSciences, Mohammed VI Polytechnic University, Lot 660–Hay MoulayRachid, 43150 Benguerir, Morocco

**Keywords:** Plant sciences, Biomarkers, Mass spectrometry

## Abstract

This study aimed at investigating the chemical composition and the hepatoprotective activities of *Plumbago indica* L. and *P. auriculata* Lam*.* LC–MS/MS analyses for the hydroalcoholic extracts of the aerial parts of the two *Plumbago* species allowed the tentative identification of thirty and twenty-five compounds from *P. indica* and *P. auriculata*, respectively. The biochemical and histopathological alterations associated with thioacetamide (TAA)-induced liver fibrosis in rats were evaluated in vivo where rats received the two extracts at three different dose levels (100, 200 and 400 mg/kg p.o, daily) for 15 consecutive days with induction of hepatotoxicity by TAA (200 mg/kg/day, i.p.) at 14th and 15th days. Results of the present study showed a significant restoration in liver function biomarkers viz*.* alanine transaminase (ALT), aspartate transaminase (AST), gamma glutamyl transferase and total bilirubin. The liver homogenates exhibited increased levels of antioxidant biomarkers: reduced glutathione (GSH) and catalase (CAT), accompanied with decline in malondialdehyde (MDA). Furthermore, treated groups exhibited a significant suppression in liver inflammatory cytokines: tumor necrosis factor-α (TNF-α) and interlukin-6 (IL-6), and fibrotic biomarker: alpha smooth muscle relaxant. Histopathological examination of the liver showed normality of hepatocytes. Noteworthy, *P. indica* extract showed better hepatoprotective activity than *P. auriculata*, particularly at 200 mg/kg. To sum up, all these results indicated the hepatoprotective properties of both extracts, as well as their antifibrotic effect was evidenced by reduction in hepatic collagen deposition. However, additional experiments are required to isolate their individual secondary metabolites, assess the toxicity of the extracts and explore the involved mechanism of action.

## Introduction

The high incidence of liver toxicity has recently been linked to many factors, the most important of which are significant exposure to high drug doses, massive metabolic activity, and the presence of numerous enzymes believed to be accountable for the generation of reactive metabolites, most notably reactive oxygen species (ROS). Toxic compounds, among them ROS, have the ability of targeting macromolecular structures or specific molecules such as nuclear receptor family members or bile acid transporters, as well as intracellular lipids, nucleic acids, or proteins. These targeted molecules have become defective units that activate secondary pathways, resulting in programmed events such as apoptosis, necrosis, and autophagy, mitochondrial failure, immunological responses, and deposition of collagen which can ultimately lead to liver fibrosis^1^.

In the development of chronic tissue injury, fibrogenesis, a dynamic process defined by the ongoing formation of fibrillar extracellular matrix (ECM), is accompanied with constant breakdown and remodeling. When deterioration is insufficient, fibrosis appears as a seemingly static consequence. The underlying diseases that induce the fibrosis cause various patterns of fibrotic development. Chronic stimulation of the wound-healing process, oxidative stress-related molecular pathways, and disruption of the so-called 'epithelial-mesenchymal' relationship resulting to reactive cholangiocytes and peribiliary fibrosis are the mechanisms responsible for fibrosis^1^. In the lack of effective hepatoprotective and antifibrotic treatments in contemporary medicine, a wide range of herbal formulations have grown in popularity for the treatment of liver disorders^2,3^.

Leadworts, the genus *Plumbago,* family Plumbaginaceae, contains about 24 flowering species and involves mostly lianas, herbs and shrubs, and often arising in saline habitats. *Plumbago* is considered the largest genus in the family with 18 species and is cultivated and utilized worldwide. Out of them*,* three species are commonly studied (*P. indica* L*.*, *P. auriculata* Lam., and *P. zeylanica* L.) due to their ornamental and medicinal values^4–6^.

*Plumbago indica* L., previously known as *P. rosea*is, is an ayurvedic, evergreen perennial shrub and grows in different regions of Europe, South-east Asia, Indonesia, Malaysia, India, China and Africa^7^. In herbal medicine, the milky juice of *P. indica* is applied in scabies and ophthalmia. A liniment prepared from bruised root mixed with bland oil is utilized in treating leucoderma, paralysis and rheumatism. The plant is also utilized to pacify numerous ailments as skin diseases, diarrhea, nervous palsy, fever, hemorrhoids, bronchitis, irritable bowel disease, amenorrhea, anemia, inflammation, and piles^8^. Various bioactive compounds were reported from the plant. These include ampelopsin-3',4',5',7-tetramethyl ether, ayanin and azaleatin, plumbaginol, plumbagin, 6-hydroxyplumbagin roseanone, droserone, zeylanone and elliptinone, myricetin-3,3',5',7-tetra methyl ether, roseanoic acid and plumbagic acid^5,6,8^.

Cape Leadwort, *P. auriculate,* is an evergreen plant grown in subtropical and tropical regions. The plant is used traditionally to treat various ailments including warts, broken bones and wounds. Powdered root was previously taken as a snuff for headaches^5^. The decoction of different plant organs of *P. auriculata* was used for the treatment of black water fever. The plant's powdered roasted root is used in scarification over fractures to accelerate healing and is applied to the body to heal stitch wounds^9^. *P. auriculata* contains numerous bioactive ingredients as azalein, capensinidin, and capensinidin 3 rhamnoside, apigenin, luteolin and their glycosides as well as plumbagin and epi‐isoshinanolone^5^.

To identify potential plant-based hepatoprotective agents with lower side effects, the secondary metabolites from *P. indica* and *P. auriculata* hydroalcoholic extracts were annotated utilizing LC–MS/MS. Then after, an in silico study was performed to dock the identified compounds into the active site of TNF-α and IL-6, two molecular targets involved in liver injury. Furthermore, the observed results were confirmed in vivo where we evaluated the hepatoprotective efficiency against TAA-induced liver toxicity in rats.

## Material and methods

### Plant materials, extraction and LC–MS/MS analysis

*Plumbago indica* and *P. auriculata* aerial parts were obtained from El-Mazhar botanical garden, Giza, Egypt after obtaining a permission to acquire the plant materials. The plants were authenticated by Mrs. Therease Labib, Consultant on Plant Identification at El-Orman Botanical Garden. Voucher specimens (Numbers: 14062020 and 15062020) were kept at the herbarium of the Department of Pharmacognosy, Faculty of Pharmacy, Cairo University. The air-dried materials (500 g, each) were subjected to exhaustive cold maceration using ethanol (70%) with frequent agitation. The extracts were separately evaporated under vacuum at 40 °C till complete dryness and furnished extraction yields of 10.8% and 9.8% for *P. indica* and *P. auriculata,* respectively.

A Thermofinnigan (Thermo Electron Corporation, USA) coupled with an LCQ-Duo ion trap mass spectrometer with an ESI source (ThermoQuest) system was utilized. The MS operated in the negative mode. The ions were detected in a full scan mode and mass range of 50–2000^10^.

### In silico investigation

To get an insight about the mechanism by which the tested extracts ameliorated the deleterious effects of TAA, we docked the most abundant compounds into the active site of TNF-α and IL-6, two molecular targets involved in liver fibrosis. The crystal structures of TNF-α (PDB id: 2AZ5) and IL-6 PDB id: 1ALU) were downloaded from protein data bank (www.pdb.org). Molecular operating environment (MOE), 2013.08; Chemical Computing Group Inc., Montreal, QC, Canada, H3A 2R7, 2016 was utilized to perform the docking analysis according to the previously described method^11^.

### In vivo investigation

#### Acute toxicity study

Acute oral toxicity was conducted according to the OECD 423 guidelines^12^. Wistar albino rats (n = 5) were randomly selected and fasted for 4 h with free access to water. Hydroalcoholic extracts of both *Plumbago* species (suspended in 0.5% NaCMC) were daily administered orally at doses 250, 500 and 1000 mg/kg for each plant extract. The animals were monitored for behavioral alterations, toxic symptoms or mortality. Both plant extracts didn’t show any toxic signs, behavior changes or mortality at the tested doses.

#### Experimental animals and design

Fifty-four adult male albino rats weighing 200–220 g were utilized in this study. They were purchased from the animal house of Faculty of Science, Cairo University. The animals were housed in separated in steel mesh cages and kept under standard conditions (ventilation, temperature (25 ± 2 °C), humidity (60–70%) and light/dark condition (12/12 h)) and fed on a standard rat pellet diet and fresh, clean drinking water. The animals were acclimatized for a period of 15 days prior to the beginning of study. Rats were randomly allocated into nine groups (6 rats each). All groups excluding group I received TAA (El-Gomhouria Company for drug and chemicals, Egypt, 200 mg/kg; i.p.) on the 14th and 15th days of the experiment. Group I; rats served as control negative group received saline on the 14th and 15th days of the experiment and vehicle (0.5% NaCMC, Sigma-Aldrich (Merck Millipore, Darmstadt, Germany) daily throughout the experiment. Group II served as control positive received two doses of thioacetamide (TAA; 200 mg/kg; i.p.) at 24 h intervals on the 14th and 15th days and also received vehicle (0.5% NaCMC) daily throughout the experiment^13^. Group III received silymarin (SLM) as standard reference drug (100 mg/kg; p.o, daily, SEDCO Pharmaceutical Industries Company, Cairo, Egypt) for 15 days and received the two doses of thioacetamide (TAA; 200 mg/kg; i.p.). Group*s* IV, V and VI rats received hydroalcoholic extract of *P. indica* aerial parts (AEPI) at oral doses of 100, 200 and 400 mg/kg/day, respectively for 15 days. Group*s* VII, VIII and IX rats received hydroalcoholic extract of *P. auriculata* aerial parts (AEPA) at oral doses of 100, 200 and 400 mg/kg/day, respectively for 15 days and received also the two doses of thioacetamide (TAA; 200 mg/kg; i.p.). Both plant extracts were assessed at three different doses similar to the same dose levels used in pervious study on another related species, *P. zeylanica* L.^14^. These dose levels were also used in order to evaluate the dose response curve for each plant. Drugs and extracts used in this experiment were suspended in 0.5% of NaCMC. Each group except the first and second groups received their corresponding treatments for fifteen days. At the end of experimental period, blood sample were withdrawn, the animals were sacrificed, and liver tissues were isolated.

#### Blood samples collection

The blood samples were withdrawn from the retro orbital vein of each rat, under mild anesthesia with phenobarbital. Blood samples were left to coagulate and centrifuged at 3000 rpm for 15 min. The sera were used to determine the activities of serum transaminases, alanine transaminase (ALT) and aspartate transaminase (AST)^15^, the level of gamma glutamyltransferace (GGT)^16^ and total bilirubin^17^ using the Biodiagnostic kits, Egypt.

#### Liver tissue homogenate

Liver specimens were rinsed with cold PBS in cold pH 7.2 and homogenated by Teflon homogenizer and then centrifuges at 3500 rpm for 15 min at −4 °C. Supernatants were utilized to determine reduced glutathione (GSH)^18^ and catalase (CAT) activities^19^ and malondialdehyde (MDA)^20^ levels as oxidative biomarkers, as well as tumor necrosis factor-α (TNF-α) and interlukin-6 (IL-6) as inflammatory biomarkers^21^ and alpha smooth muscle relaxant (α-SMA)^22^ as fibrotic biomarker using appropriate commercial kits.

#### Histological and histochemical assessment studies

The Livers were dissected immediately after death and the specimens were fixed in neutral-buffered formalin saline (10%, at least 72 h). All the specimens were washed in tap water for 30 min and dehydrated in ascending grades of alcohol, cleared in xylene, and embedded in paraffin. Serial sections of 4–5 µm thick were cut and stained with haematoxylin and eosin for histopathological investigation^23^.

### Statistical analysis

The significant differences between the means of the groups (mean ± standard error of mean) were analyzed with one-way analysis of variance (ANOVA) followed by the Tukey's post hoc multiple comparisons test. Results were considered as statistically significant when *p* < 0.05. The statistical analysis and figures were generated using GraphPad Prism 8.4.3.

## Results

### Phytochemical analyses

Profiling of the phenolic metabolites in the hydroalcoholic extracts of *P. indica* and *P. auriculata* aerial parts were performed by LC–MS/MS. In total, thirty and twenty-five secondary metabolites were detected in *P. indica* and *P. auriculata*, respectively. Twenty-one compounds were common in both extracts. The compounds were identified and tentatively annotated based on their retention times, molecular weight, and fragmentation pattern as well as comparison with reported data found in literature and online data base (Mass Bank) (Table 1).

**Table 1:** Phenolic compounds of the hydroalcoholic extracts of the aerial parts of *Plumbago indica* and *P. auriculata* by LC–MS–MS.

No	Compound name	Rt	*m/z*	Relative abundance (%)	
				*P. indica*	*P. auriculata*
1	Gallic acid^3^	1.61	169	6.82	1.31
2	Caffeic acid	7.08	179, 163, 107	3.94	7.20
3	Catechin^10^	7.51	289, 275	4.37	2.05
4	Syringic acid^5^	7.77	197, 153	6.97	1.15
5	Coumaric acid	8.31	163, 159	0.81	0.82
6	Cinnamic acid	8.51	147, 119	5.64	0.34
7	Ellagic acid^10^	8.87	301, 229, 211	9.68	0.55
8	Methyl gallate^10^	9.90	183, 171,	2.92	50.36
9	Chlorogenic acid	11.58	353, 191, 135, 85	1.32	1.13
10	Rutin^10^	12.55	609, 301	3.14	1.93
11	Callistephin	13.20	269	–	3.50
12	Vitexin^10^	13.23	431, 341, 311	5.99	–
13	Resveratrol	16.62	227, 185, 143	–	1.25
14	Ferulic acid	17.42	193, 165	2.50	5.35
15	Quercetin-3,7-O-alpha-L-dirhamnopyranoside	18.45	446, 301	3.31	–
16	Kaempferitrin	19.63	430, 285,	1.94	–
17	Gentistein 8-C-glucoside	19.91	311, 283	1.46	4.11
18	Pyro catechol^5^	21.32	109	2.75	4.77
19	Quercetin-3-glucuronide	23.68	477, 301	2.72	–
20	Kaempferol glucoside	26.28	447, 285, 93	5.66	3.47
21	Tricin glucoside	26.89	491, 329, 299	4.72	–
22	Daidzin	27.05	415, 253	2.65	–
23	3-Hydroxy-3’, 4’, 5’,-trimethoxyflavone	27.65	327, 297, 210	6.73	2.65
24	Plumbagin^6^	29.54	187	4.88	0.35
25	Luteolin-7-glucoside	30.77	285, 447	1.05	0.73
26	Puerarin	30.65	415, 277, 235	0.00	0.70
27	Apigenin glucoside^3^	30.75	431, 269, 225	0.00	0.68
28	Myricetin	30.85	317, 179	0.44	0.00
29	Quercetin-3-O-α-L-rahmnopyranoside^5,10^	31.11	447, 301	1.50	–
31	Quercetin-3-O-β-D-glucopyranoside^6,10^	31.31	463, 301, 179	0.78	–
32	Plumbagin *5-O-α-L-*rhamnopyranoside^6^	31.40	333, 187, 159	0.36	–
33	6-Ethoxy-3(4’-hydroxyphenyl)-4-methylcoumarin	31.58	295, 266, 238	2.84	1.46
34	Vanillin^5^	31.69	151, 109	0.45	1.36
35	Naringenin^5^	31.78	271, 177, 151	1.48	0.88

RT. (min): Retention time per minute.

M/Z: mass fragmentation.

### Pharmacological analyses

#### Effect of pretreatment with hydroalcoholic extracts of the aerial parts of *P. indica* or *P. auriculata* on serum hepatic functions biomarkers in TAA-induced liver toxicity in rats

Injection of TAA significantly elevated serum ALT, AST, GGT, and bilirubin levels compared to normal control group values by 3.71, 3.57, 5.74, and 4.99 folds, respectively. Both plants-treated groups significantly reduced the elevated levels of serum liver biomarkers compared to TAA group. Pretreatment with *P. indica* showed remarkable amelioration of the elevation of ALT, AST, GGT, and bilirubin levels by about 26.69%, 27.61%, 33.24% and 31.19%, respectively at a dose of 100 mg/kg, by about 63.85%, 55.39%, 57.72% and 61.12%, respectively at 200 mg/kg, and by about 42.91%, 42.38%, 45.42% and 56.35%, respectively at 400 mg/kg compared to TAA group. *Plumbago auriculata* treated groups controlled the rising of ALT, AST, GGT, and bilirubin levels at a dose of 100 mg/kg by about 19.88%, 21.40%, 24.16% and 29.05%, respectively at a dose of 200 mg/kg by about 50.45%, 46.87%, 48.59% and 57.80%, respectively and at a dose of 400 mg/kg by about 37.00%, 35.43%, 40.55% and 39.74%, respectively when compared to TAA group. Treatments of rats with both plant extracts before TAA administration showed an improvement in liver function versus TAA group. These observed results were comparable to those of the reference drug, silymarin (Table 2).

**Table 2:** Effect of pretreatment with hydroalcoholic extracts of the aerial parts of *P. indica* or *P. auriculata* on serum hepatic functions biomarkers in TAA-induced liver toxicity in rats.

Parameters	ALT (U/L)	AST(U/L)	GGT(U/L)	Bilirubin (umol/L)
Control	32.35 ± 0.51	34.44 ± 1.7	5.93 ± 0.21	6.09 ± 0.11
TAA	120.04 ± 2.52*	123.02 ± 3.11*	34.06 ± 2.55*	30.40 ± 1.60*
TAA + SLM (100 mg/Kg)	41.09 ± 3.62^@^	43.32 ± 3.4^@^	7.76 ± 0.56^@^	7.77 ± 0.7^@^
TAA + AEPI (100 mg/Kg)	87.99 ± 4.81^@^*	89.05 ± 4.13^@^*	22.74 ± 1.66^@^*	20.92 ± 1.93^@^*
TAA + AEPI (200 mg/Kg)	43.39 ± 3.31^@^	54.88 ± 3.37^@^*	14.40 ± 0.58^@^*	11.82 ± 1.07^@^*
TAA + AEPI (400 mg/Kg)	68.53 ± 1.99^@^*	70.89 ± 2.33^@^*	18.59 ± 0.96^@^*	13.27 ± 0.83^@^*
TAA + AEPA (100 mg/Kg)	96.18 ± 5.72^@^*	96.70 ± 4.89^@^*	25.83 ± 0.74^@^*	21.57 ± 0.84^@^*
TAA + AEPA (200 mg/Kg)	59.48 ± 1.43^@^*	65.36 ± 2.89^@^*	17.51 ± 0.72^@^*	12.83 ± 0.75^@^*
TAA + AEPA (400 mg/Kg)	75.62 ± 2.7^@^*	79.44 ± 2.77^@^*	20.25 ± 1.8^@^*	18.32 ± 1.06^@^*

Results were expressed as Mean ± S.D., *n* = 5. SLM: Silymarin, *^,@^Significantly different from normal control and TAA groups at *p* > 0.05, respectively.

#### Effect of pretreatment with hydroalcoholic extracts of the aerial parts of *P. indica* or *P. auriculata* on the hepatic oxidative stress biomarkers in TAA-induced liver toxicity in rats

**Figure 1:**
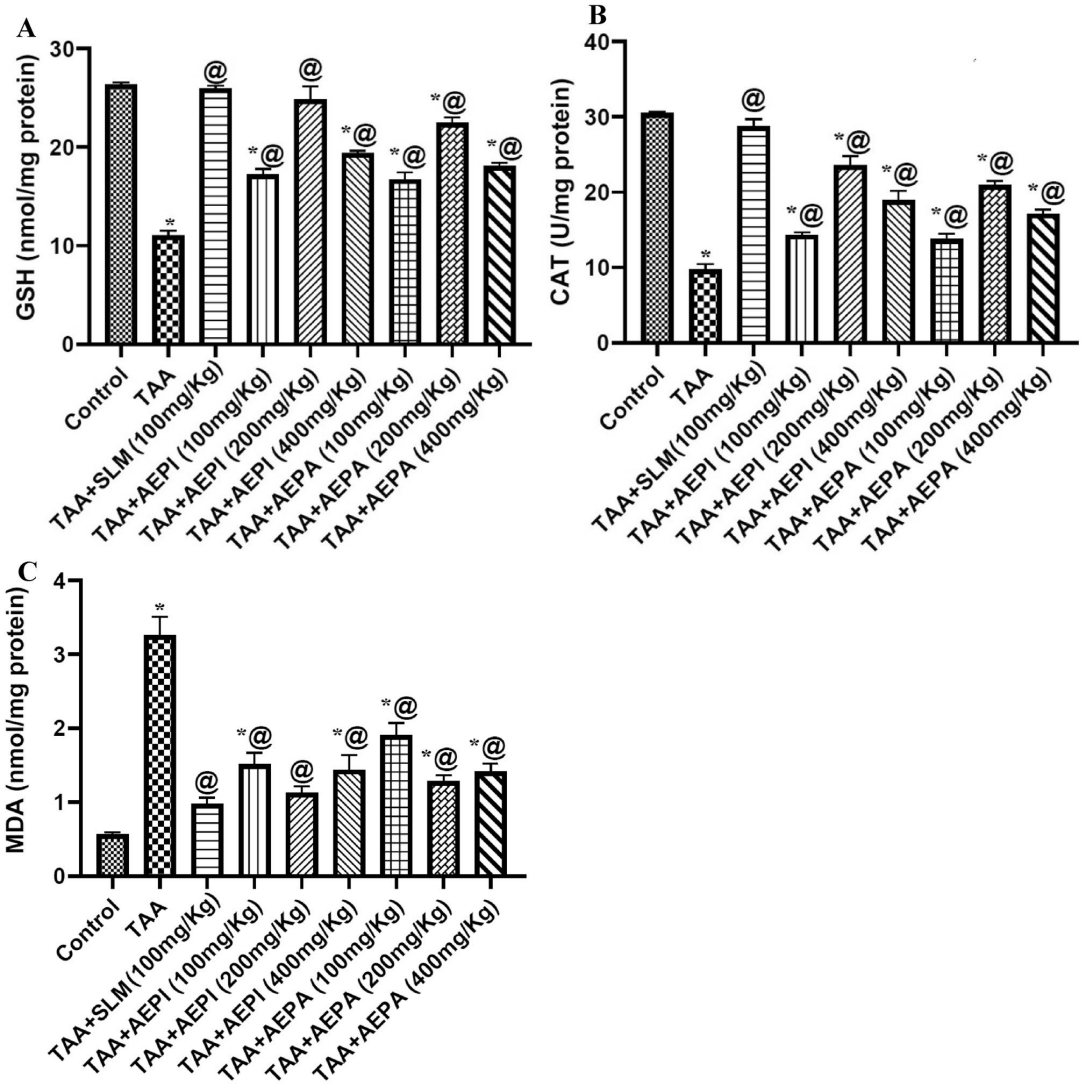
Effects of pretreatment with hydroalcoholic extracts of the aerial parts of *P. indica* or *P. auriculata* on the liver contents of oxidative stress markers in TAA-induced liver toxicity in rats. (**A**) reduced glutathione (GSH), (**B**) catalase enzyme (CAT) and (**C**) malondialdehyde (MDA). Animals were given TAA (200 mg/kg/day; i.p.) on 14th and 15th days. Other regimens were given orally and daily for 15 days. Results were expressed as Mean ± SD, *n* = 5. SLM: Silymarin, *^,@^Significantly different from normal control and TAA groups at *p* < 0.05, respectively.

Induction of liver injury by TAA significantly decreased in antioxidant enzymes evidenced by GSH levels by about 58.17% and CAT levels by about 67.85%, as well as a significant elevation in lipid peroxidation that was measured by MDA values to 5.7 fold of a thiobarbituric acid reactive substance as compared to normal group. On the other hand, Administration of *P. indica* hydroalcoholic extract at different doses, 100, 200, and 400 mg/kg, significantly increased the lever contents of GSH levels about 56.25%, 125.11% and 75.92%, respectively and CAT levels by about 46.43%, 140.92% and 93.82%, respectively while the values of MDA was significantly decreased by about 53.50%, 65.34% and 55.83%, respectively, compared to TAA control group. Doses of *P. auriculata* hydroalcoholic extract, 100, 200, and 400 mg/kg also controlled the reduction of GSH by about 51.77%, 103.72%, and 64.19% and CAT by about 41.12%, 113.78% and 75.0% respectively. On the same way, doses of *P. auriculata,* 100, 200 and 400 mg/kg, also hindered the risings of MDA values by about 41.41%, 60.49% and 56.58%, respectively as compared to TAA group. Noteworthy, all results matched with those obtained by pretreatment with silymarin that restored the reduction of GSH and CAT by about 135.54% and 193.88%, respectively as well as restrict the MDA elevation by about 70.0% (Fig. 1).

#### Effects of pretreatment with hydroalcoholic extracts of the aerial parts of *P. indica* or *P. auriculata* on the hepatic inflammatory biomarkers in TAA-induced injury in rats

**Figure 2:**
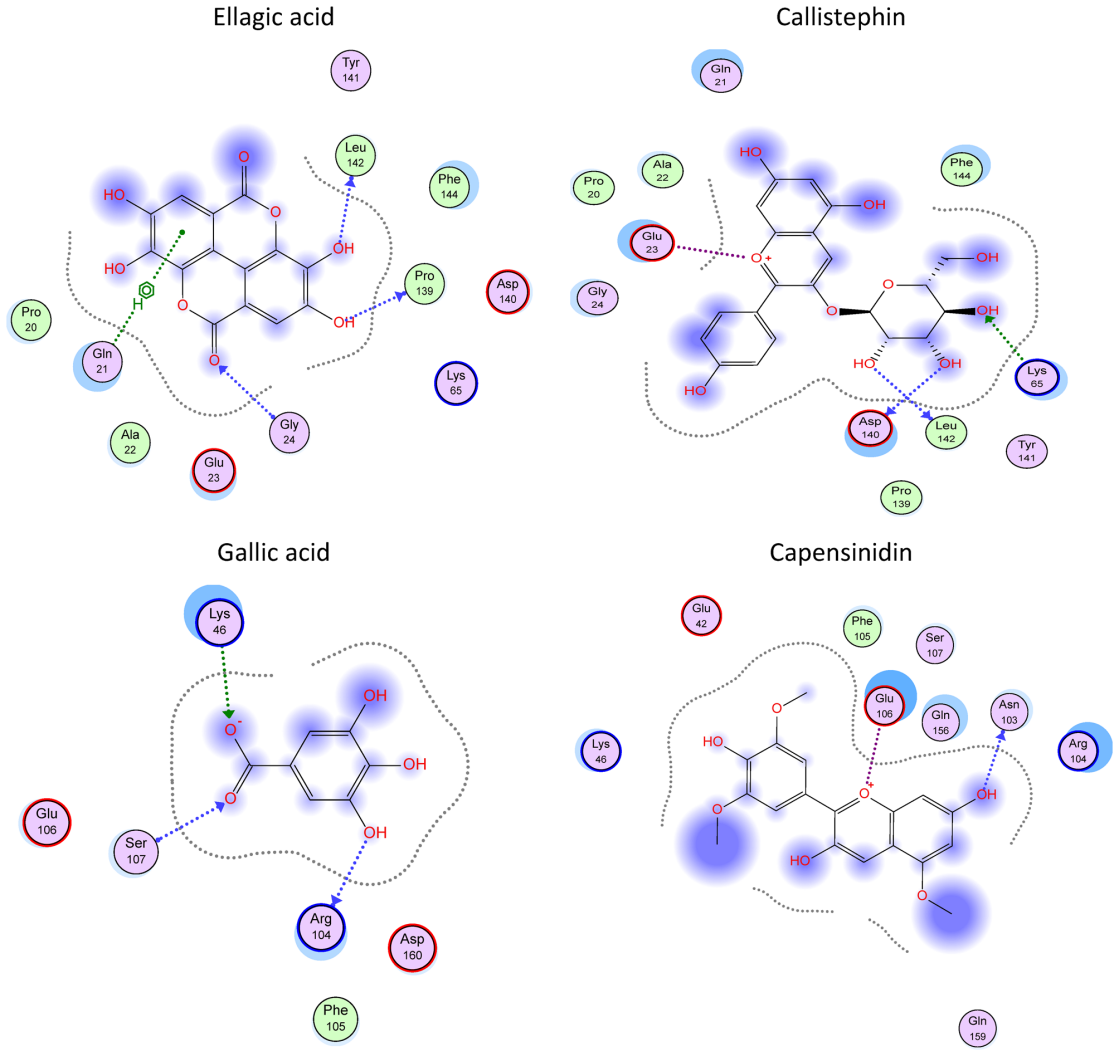
2D representative of selected secondary metabolites of *P. indica* and *P. auriculata* in TNF-α and IL-6 active sites using in silico modeling.

Initially, we virtually explored the interactions of the individual secondary metabolite from both extracts with the active site of both hepatic inflammatory biomarkers (TNF-α and IL-6). The docked compounds furnished considerable binding energies and several hydrogen bonding and hydrophobic and ionic interactions, Table 3 and Fig. 2.

**Table 3:** Score functions and interactions of *P. indica* and *P. auriculata* secondary metabolites in TNF-α and IL-6 active sites using in silico modeling.

Compound	TNF-α		IL-6	
	SF*	Interactions	SF*	Interactions
18	− 5.92	Glu 23 (H bonding)	− 7.34	Arg 104 (H bonding)
1	− 8.41	Lys 65 (Salt bridge) Phe 144 (Hydrophobic interaction)	− 13.72	Arg 104 (H bonding) Lys 46 (Salt bridge) Ser 107 (H bonding)
35	− 7.91	Glu 23 (H bonding) Lys 65 (H bonding)	− 10.08	Glu 106 (H bonding) Asp 160 (H bonding)
8	− 7.08	Glu 23 (H bonding) Ala 22 (H bonding)	− 13.48	Arg 104 (H bonding) Ser 107 (H bonding)
25	− 6.04	Glu 23 (H bonding)	− 8.68	Glu 106 (H bonding)
4	− 7.32	Lys 65 (H bonding and salt bridge)	− 12.05	Lys 45 (Salt bridge) Ser 107 (H bonding)
36	− 8.20	Lys 65 (H bonding and hydrophobic interaction) Asp 140 (hydrophobic interaction)	− 10.54	Asp 160 (H bonding through solvent) Arg 104 (H bonding through solvent) Thr 163 (H bonding through solvent)
13	− 7.36	Glu 23 (H bonding) Gly 24 (H bonding) Lys 65 (hydrophobic interaction)	− 11.21	Asp 160 (H bonding) Ser 108 (H bonding through solvent) Glu 42 (H bonding through solvent)
33	− 6.77	Ala 22 (H bonding)	− 6.52	Glu 106 (Hydrophobic interaction)
23	− 5.61	Asp 140 (hydrophobic interaction)	Failed	
12	− 8.74	Glu 23 (H bonding) Ala 22 (H bonding) Lys 65 (H bonding) Gln 21 (H bonding)	− 12.46	Glu 42 (H bonding through solvent) Ser 108 (H bonding through solvent) Glu 106 (H bonding)
28	− 7.06	Glu 23 (H bonding)	− 10.69	Asp 160 (H bonding) Glu 106 (H bonding)
10	− 9.39	Lys 65 (H bonding) Glu 23 (H bonding) Asp 140 (H bonding)	− 12.51	Arg 104 (H bonding) Glu 106 (H bonding) Gln 159 (H bonding through solvent) Gln 156 (H bonding through solvent)
17	− 8.67	Glu 23 (H bonding) Gly 24 (H bonding) Ala 22 (H bonding)	− 10.48	Ser 107 (H bonding)
27	− 8.50	Glu 23 (H bonding) Gly 24 (H bonding) Lys 65 (hydrophobic interaction)	− 10.23	Glu 42 (H bonding through solvent) Ser 108 (H bonding through solvent)
7	− 9.65	Gly 24 (H bonding Pro 139 (H bonding) Gln 21 (Hydrophobic interaction) Leu 142 (H bonding)	− 11.63	Glu 106 (H bonding through solvent) Ser 107 (H bonding through solvent)
31	− 9.56	Glu 23 (H bonding) Leu 142 (H bonding) Lys 65 (H bonding)	− 12.25	Arg 104 (H bonding) Asp 160 (H bonding through solvent)
11	− 9.83	Glu 23 (H bonding) Lys 65 (H bonding) Leu 142 (H bonding) Asp 140 (H bonding)	− 12.29	Arg 104 (H bonding) Glu 106 (Salt bridge) Gln 159 (H bonding through solvent)
Plumbagic acid	− 8.25	Glu 23 (H bonding) Lys 65 (H bonding)	− 12.70	Thr 43 (H bonding) Asp 160 (H bonding) Lys (Hydrogen bonding and hydrophobic interaction)
Azalein	− 9.13	Glu 23 (H bonding) Ala 22 (H bonding) Pro 20 (H bonding)	− 11.80	Glu 106 (Hydrophobic interaction)
Azaleatin	− 8.43	Glu 23 (H bonding) Gly 24 (H bonding) Pro 139 (H bonding)	− 11.33	Arg 104 (H bonding) Glu 106 (Hydrophobic interaction)
Luteolin	− 8.24	Lys 65 (H bonding)	− 11.24	Asn 103 (H bonding) Glu 106 (Hydrophobic interaction)
Ayanin	− 8.23	Lys 65 (H bonding)	− 10.44	Arg 104 (H bonding)
Capensinidin	− 9.18	Glu 23 (H bonding) Pro 139 (H bonding)	− 13.67	Glu 106 (Salt bridge) Asn 103 (H bonding)
Droserone	− 8.39	Lys 65 (Salt bridge)	− 12.88	Asp 160 (H bonding)

Compound numbers are from Table 1.

*Score function (kcal/mol).

**Figure 3:**
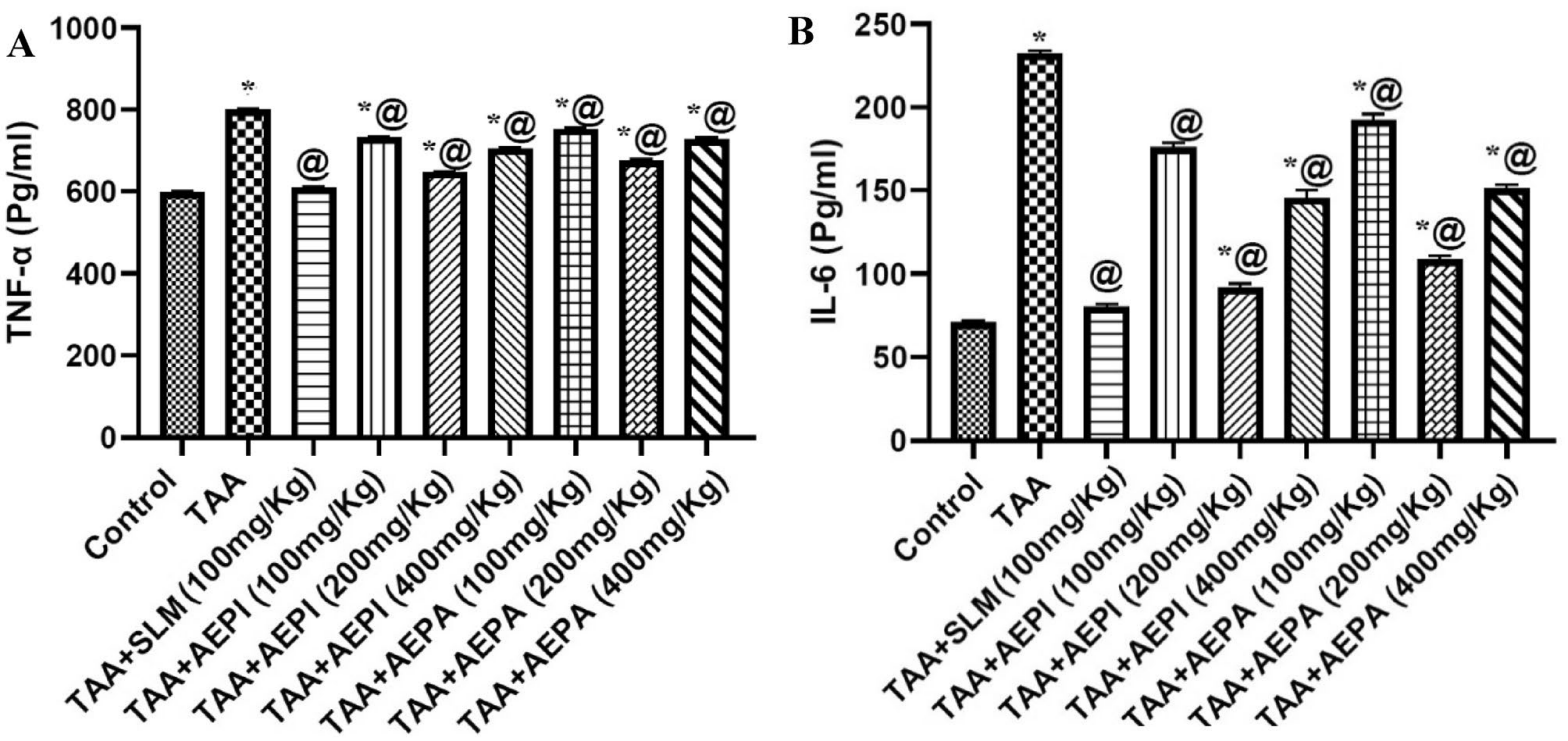
Effects of pretreatment with hydroalcoholic extracts of the aerial parts of *P. indica* or *P. auriculata* on the hepatic contents of inflammatory markers in TAA-induced injury in rats. (**A**) Tumor necrosis factor (TNF-α) and (**B**) interleukin (IL-6). Animals were given TAA (200 mg/kg/day; i.p.) on 14th and 15th days. Other regimens were given orally and daily for 15 days. Results were expressed as Mean ± SD, *n* = 5. SLM: Silymarin, *^,@^Significantly different from normal control and TAA groups at *p* < 0.05, respectively.

These results were further confirmed in vivo where injection of TAA significantly elevated liver TNF-α and IL-6 by 1.33 and 3.01 fold, respectively compared to normal group values. Doses of the hydroalcoholic extract of *P. indica* aerial parts (100, 200 and 400 mg/kg) significantly decreased the raised TNF-α levels by about 8.43%, 19.04% and 11.93%, respectively, as well as IL-6 levels by about 24.44%, 60.54% and 37.40%, respectively compared to TAA control group. The rats pretreated with the hydroalcoholic extracts of *P. auriculata* aerial parts at doses of 100, 200 and 400 mg/kg showed remarkable reductions in the elevated TNF-α by about 5.92%, 15.57% and 9.12%, respectively and IL-6 by about 17.21%, 53.25% and 35.01%, respectively, compared to TAA control groups. Both extracts showed an obvious reduction in TNF-α and IL-6 levels. Moreover, pretreatment with silymarin showed a reduction by about 23.78% and 65.55% for TNF-α and IL-6 levels, respectively (Fig. 3).

#### Effect of pretreatment with hydroalcoholic extracts of the aerial parts of *P. indica* or *P. auriculata* on hepatic α-SMA in TAA-induced liver injury in rats

**Figure 4:**
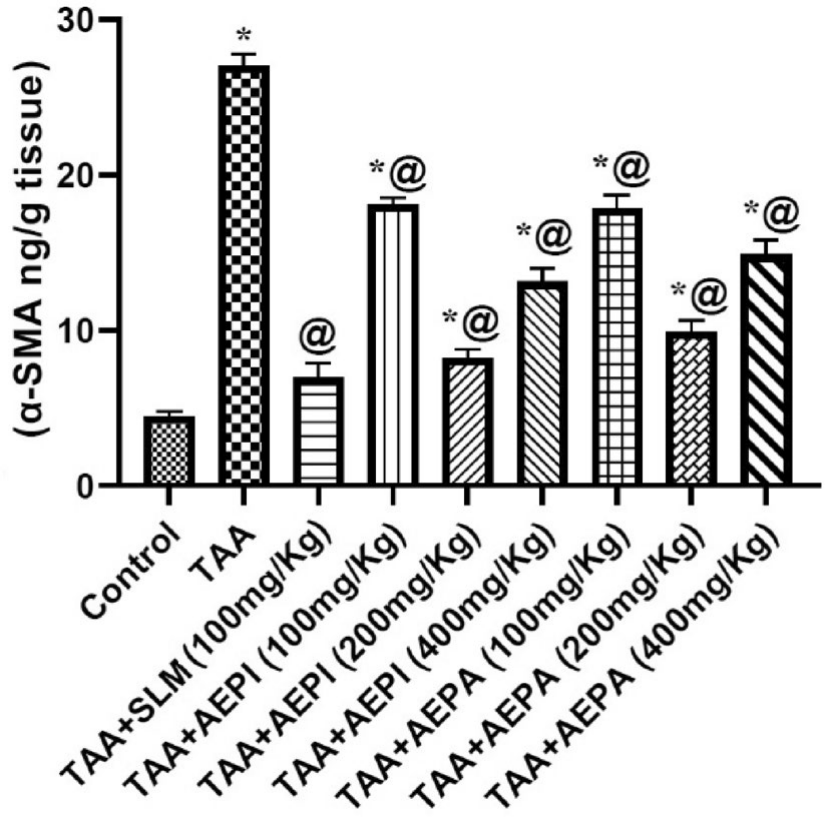
Effects of pretreatment with hydroalcoholic extracts of the aerial parts of *P. indica* or *P. auriculata* on the hepatic α-SMA in TAA-induced liver injury in rats. Animals were given TAA (200 mg/kg/day; i.p.) on 14th and 15th days. Other regimens were given orally and daily for 15 days. Results were expressed as Mean ± SD, *n* = 5. SLM: Silymarin, *^,@^Significantly different from normal control and TAA groups at *p* < 0.05, respectively.

Induction of liver dysfunction in rats using TAA significantly provoked the hepatic α-SMA by 6.04 fold as compared to the normal group (Fig. 4). Both plant extracts revealed a noticeable protection against the elevation of α-SMA by about 33.13%, 69.61% and 51.45% for 100, 200 and 400 mg/kg of *P. indica* extract*,* respectively and 33.79%, 63.44%, and 44.95% for 100, 200 and 400 mg/kg of *P. auriculata* extract, respectively compared to TAA group. In spite of this observable protection accompanied with both plant extracts against fibrosis which is comparable by the protection showed by silymarin, there is still different in α-SMA values compared with control group indicating an antifibrotic effect.

### Histopathological examination of liver tissue

**Figure 5:**
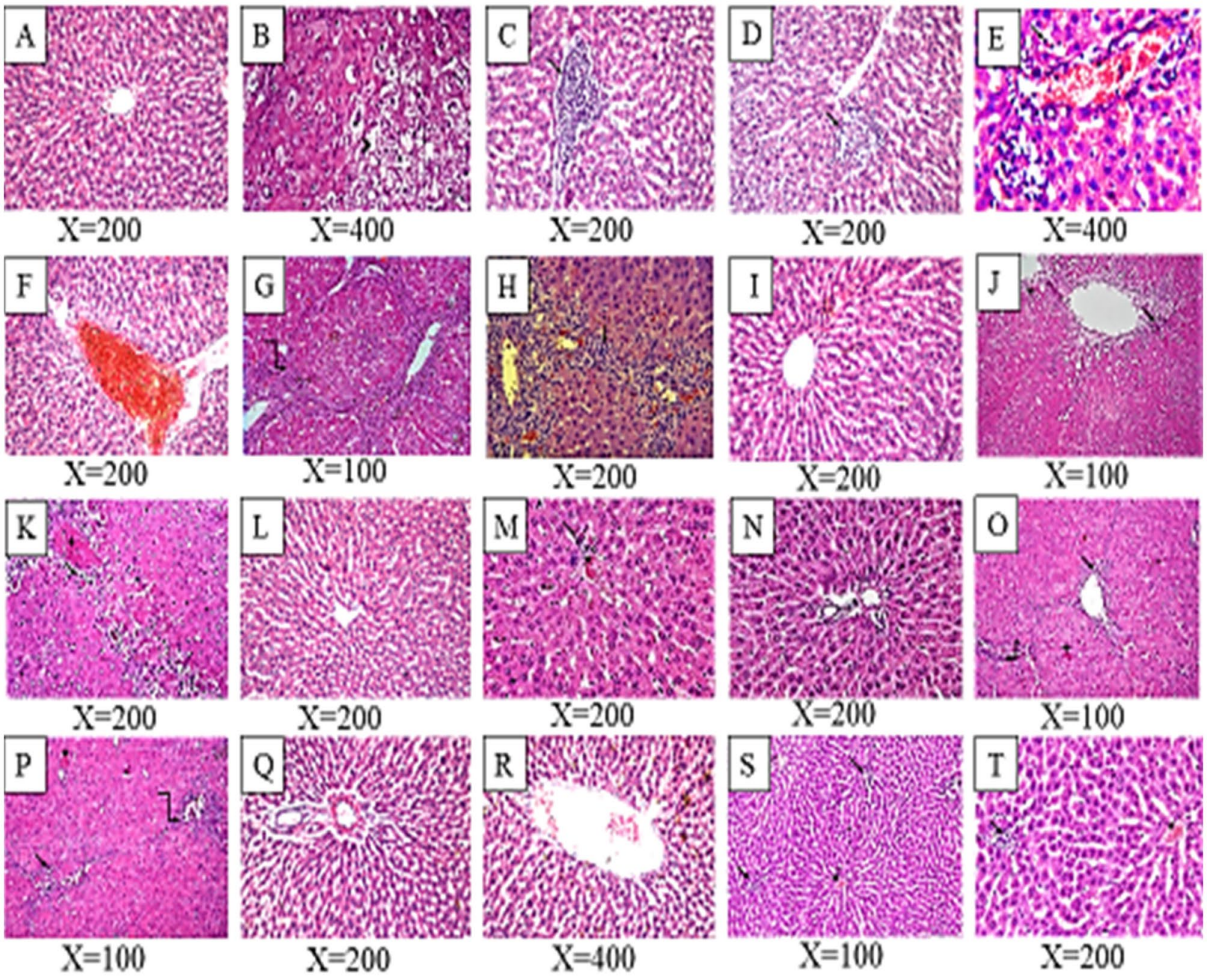
Histopathological findings of liver sections for different studied groups. (**A**) Section of normal control group; (**B–H**) Sections of TAA (200 mg/kg bw/i.p.) treated group; (**I**) Sections of silymarin (100 mg/kg bw/p.o.) treated group; (**J**, **K**) Sections of *P. indica* (100 mg/kg bw/p.o) treated rats; (**L**) Section of *P. indica* (200 mg/kg bw/p.o.) treated rats; (**M**, **N**) Sections of *P. indica* (400 mg/kg bw/p.o.) treated rats; (**O, P**) Sections of *P. auriculata* (100 mg/kg bw/p.o.) treated rats; (**Q**, **R**) Sections of *P. auriculata* (200 mg/kg bw/p.o.) treated rats; (**S**, **T**) Sections of *P. auriculata* (400 mg/kg bw/p.o.) treated rats. (Arrowhead, vacuolar degeneration; four point star, cellular apoptosis; Arrow pointed to inflammatory cells; five point star, vascular congestion; elbow arrow connector, fine strands of fibrous tissue, H&E stained).

In regards to the histopathological examination, there was no histopathological alteration and the normal histological structure of the central vein and surrounding hepatocytes in the parenchyma were detected in normal control rats (Fig. 5A) while, examination of the livers of TAA rats revealed swelling besides vacuolar degeneration observed in hepatocytes (Fig. 5B) and associated with focal mononuclear leucocytes inflammatory cells aggregation in the hepatic parenchyma (Fig. 5C). The hepatic parenchyma showed also focal necrosis, as well as congestion in the portal and central veins (Fig. 5D–F). Collagen fibers deposition was noticed around central vein and portal tract besides pseudolobulation of hepatocytes with fibroblasts was also observed (Fig. 5G, H). Normal hepatic architecture was observed at silymarin treated group (Fig. 5I). Rats protected by *P. indica* (100 mg/kg) showed fibrosis in the portal area of liver and few inflammatory cells with many apoptotic cells (Fig. 5J) and parenchymal cells with marked apoptosis was also observed (Fig. 5K). Much better improvement was detected in *P. indica* group (200 mg/kg), as there was a normal hepatic architecture observed (Fig. 5L) while *P. indica* (400 mg/kg) treated group showed also normal parenchymatous architecture of the liver but there is a little cellular infiltration, inflammatory cell and vascular congestion were noted (Fig. 5M, N). Some livers of *P. auriculata* administrated rats at the dose of 100 mg/kg showed fine strands of fibrous tissue extending from portal areas toward the parenchyma (Fig. 5O) and the hepatic parenchymal cells are near to the normal appearance with mild vacuolation (Fig. 5P). Liver sections of rats treated by *P. auriculata* (200 mg/kg) signifying that the portal area and surrounding hepatocytes in the parenchyma was histological normal (Fig. 5Q), but the central veins and sinusoid were dilated (Fig. 5R) while preserved liver architecture against damage was observed but small foci of inflammatory cells were noted in some fields at the rats protected by *P. auriculata* (400 mg/kg) (Fig. 5S, T).

## Discussion

In the current study, phytochemical analyses by LC–MS/MS were performed in order to investigate the bioactive compounds present in of *P. indica* and *P. auriculata*. LC–MS/MS analysis revealed the presence of numerous compounds that was tentatively identified for the first time in both plants such as genistein 8-C-glucoside, 3-hydroxy-3',4',5'-trimethoxyflavone, 6-ethoxy-3(4'-hydroxyphenyl)-4-methylcoumarin and luteolin. The findings of LC–MS/MS analyses also in parallel with previous reports that revealed the presence of plumbagin in both plants^6^.

The current study also investigated the protective role of *P. indica* and *P. auriculata* hydroalcoholic extracts on liver injury induced by TAA-administration in male albino Wistar rats. To the best of our knowledge, this is the first *in-vivo* assessment for the anti-inflammatory and anti-fibrotic activities of both plants against TAA-induced fibrosis in rats. TAA is a well-established model for induction of hepatic fibrosis by producing free radicals during its metabolism resulting in oxidative stress mediated acute hepatic inflammation and destruction of hepatocytes in the liver^24–26^. Thioacetamide metabolites covalently bind to the liver macromolecules causing dramatic elevation of serum levels of ALT, AST, GGT, and bilirubin, a decrease in antioxidant capacity as proved by depletion of GSH and CAT levels and an increase in lipid peroxidation as confirmed by elevation of MDA levels and by increasing ROS production. ROS are shown to enhance pro-inflammatory cytokines production (TNF-α and interleukin-6 (IL-6)). There are also solid links between inflammation, oxidative stress, fibrogenesis and angiogenesis^27^. Liver fibrosis induced by TAA is characterized by several amendments occurred at hepatic ECM^28^.

The outcomes of the present investigation showed that pretreatment of TAA-induced hepatic fibrotic rats with *P. indica* extract at 200 mg/kg exhibited a pronounced protection on the levels of serum hepatic enzymes, antioxidant, anti-inflammatory and anti-fibrotic marker: α-SMA, against the toxic and fibrotic changes induced by TAA. The current study also revealed that the hydroalcoholic extract of *P. indica* aerial parts has a potency similar to the root alcoholic extract of the same plant that was assessed previously against the same hepatotoxic agent, TAA^29^. Moreover, administration of both extracts ahead of TAA reduced the elevated enzyme levels in serum plasma resulting from the stabilization of these biomarkers, clearly showing a preventive effect of the plants on TAA intoxication. Reduced levels of GSH have been associated with TAA induced hepatitis and are closely correlated to the lipid peroxidation and disturbance of calcium ions induced by toxic agents^30,31^. In extracts-treated groups, there was an observed boost in tissue GSH content showing that both extracts tend to reverse the tissue depletion of GSH in hepatic tissues. The hydroalcoholic extracts of both plants cause a significant increase in hepatic CAT activities and, thus, diminishes the oxidative injury in the liver due to the free radical establishment by the action of TAA.

Lipid peroxidation is considered as one of the important characteristics of oxidative stress^32^. Rats treated with TAA showed significantly elevated levels of lipid peroxidation, which is characterized by increase in the levels of MDA resulted in the failure of the antioxidant defense mechanism^33^. There was a decrease in the levels of MDA in plants-treated rats previously to their intoxication with TAA, showing that both extracts may exert a preventive action on hepatic tissue. In case of silymarin, this compound also exerted antioxidant activities indicated by prevention of GSH and CAT depletion besides maintain MDA at normal levels. Consistent with these data, Eldhose et al.^29^ was previously reported that silymarin have a clear antioxidant activity at 100 mg/kg against TAA-induced liver injury. Furthermore, a chronic subjection of rats to TAA resulted in an increase in hepatic TNFα and IL-6 levels. This is in parallel with previous studies^26,34^. Silymarin also revealed anti-inflammatory potential where it decreased hepatic TNF-α and IL-6 contents which is in line with a previous study^35^.

The anti-inflammatory action of both plants also observed as a reduction in hepatic TNF-α and IL-6 levels compared to TAA group. Here, induction of liver fibrosis via TAA increased liver α-SMA that comes in agreement with several previous studies^36,37^. α-SMA is an actin isoform and a specific marker for smooth muscle cell differentiation^38^. Silymarin, on the other, demonstrated anti-fibrotic activities in rats evidenced by significant reduction in hepatic α-SMA. Similar results were reported where daily administration of silymarin resulted in modulation of fibrosis by reducing the hepatic α-SMA contents^39,40^.

Numerous studies have highlighted the hepatoprotective and antioxidants effects of several *Plumbago* species^41,42^*.* Rajasekaran and Periasamy^43^ studied the protective potential of ethanolic root extract of *P. indica* on paracetamol-induced liver damage at a dose of 200 and 400 mg/kg of the extract. Eldhose et al.^29^ has also described the curative effect of *P. indica* roots using its methanolic extracts at different doses, 100 and 200 mg/kg bw, on TAA-induced liver damage in Wistar albino rats. Moreover, *P. auriculata* was proven to have an antioxidant capacity verified by several assessments such as oxygen radical absorbance capacity (ORAC), the thiobarbituric acid reactive substances (TBARS), ferric reducing antioxidant power (FRAP), and the nitro-blue tetrazolium (NBT)^44^. The results of current study suggested that both *Plumbago* species furnished marked hepatoprotective properties owing to their antioxidant, antiinflammatory and antifibrotic effects against TAA induced liver toxicity.

## Conclusion

The result of the current work highlighted that pretreatment of *P. indica* or *P. auriculata* hydroalcoholic extracts have significant hepatoprotective effects on TAA-induced liver oxidative stress and liver fibrosis in rats through their robust antioxidant and anti-inflammatory potentials. Both doses of *P. indica* and *P. auriculata* at 200 mg/kg showed promising protection against TAA-induce liver fibrosis with superior hepatoprotective activity accounted for *P. indica*. The bioactive compounds present at both plants, identified by LC–MS/MS, could be responsible for these activities. However, further clinical studies are needed in order to validate the usage of these extracts as hepatoprotective supplements, to ameliorated fibrosis and thus could be used as antifibrotic agents.

### Institutional review board statement

The design of the experiments and the protocol conforms the guidelines of the National Institute of Health (NIH) and National Research Center- Medical Research Ethics Committee for the use of animal subjects and approved by ethical research committee of Faculty of Pharmacy, Cairo University (Approval Numbered N-14052020). The study was carried out in compliance with ARRIVE guidelines.

## Data Availability

All data generated and analyzed during this study are included in this published article.
